# Effects of Magnetic Nanoparticles on the Functional Activity of Human Monocytes and Dendritic Cells

**DOI:** 10.3390/ijms24021358

**Published:** 2023-01-10

**Authors:** Marta Donini, Francesca Pettinella, Giorgia Zanella, Salvatore Calogero Gaglio, Carlo Laudanna, Monica Jimenez-Carretero, Concepcion Jimenez-Lopez, Massimiliano Perduca, Stefano Dusi

**Affiliations:** 1 Section of General Pathology, Department of Medicine, University of Verona, Strada Le Grazie 8, 37134 Verona, Italy; 2 Department of Biotechnology, University of Verona, Strada Le Grazie 15, 37134 Verona, Italy; 3 Department of Microbiology, Faculty of Sciences, University of Granada, 18071 Granada, Spain

**Keywords:** monocytes, dendritic cells, magnetic nanoparticles, pro-inflammatory mediators, reactive oxygen species, inflammation, immunity

## Abstract

The use of nanoparticles in medicine is sometimes hampered by their potential to activate immune cells, eliciting inflammation or allergy. We investigated whether magnetic nanoparticles (MNPs) or biomimetic magnetic nanoparticles (BMNPs) affect relevant activities of human monocytes. We found that the nanoparticles neither elicited the production of pro-inflammatory mediators IL-6 and TNFα by resting monocytes (when BMNP dose < 300 μg/mL) nor enhanced their secretion induced by R848, a molecule engaging virus-recognizing receptors, or bacterial lipopolysaccharide (LPS). MNPs and BMNPs neither induced the generation of reactive oxygen species (ROS), nor affected the ROS production elicited by the NADPH oxidase activator phorbol myristate acetate (PMA) or the fungal derivative β-glucan. BMNPs, but not MNPs, caused an up-regulation of the maturation markers CD80, CD83, and CD86 in immature monocyte-derived dendritic cells (DCs), whereas both nanoparticles did not affect the LPS-induced expression of these markers. Moreover, the nanoparticles were greedily ingested by monocytes and DCs without altering their viability. Therefore, these nanoparticles are candidates for medical applications because they do not activate pro-inflammatory activities of monocytes. Furthermore, their ability to stimulate DC maturation could be used for the design of vaccines. Moreover, harmlessly engulfed nanoparticles could be vehicles to carry molecules inside the immune cells to regulate the immune response.

## 1. Introduction

Magnetic nanoparticles (MNPs) have emerged in recent years as a powerful tool as directed chemotherapy and/or hyperthermia agents in the biomedical field [1,2,3]. Among iron oxide nanoparticles, MamC-mediated biomimetic magnetic nanoparticles (BMNPs), synthetized inorganically in the presence of MamC protein (magnetosome membrane-associated protein from *Magnetococcus marinus* MC-1), have emerged as a better candidate in this context, offering advantages over traditional MNPs. BMNPs are produced by taking inspiration from nature, in particular, from magnetosome magnetite produced by magnetotactic bacteria. MamC controls magnetite nucleation and growth by exerting both an ionotropic effect (electrostatically binding Fe cations by acidic amino acids), and a template effect for magnetite growth [4]. This control results in novel biomimetic nanoparticles (BMNPs) that are different than those produced in protein-free experiments (MNPs). On top of being superparamagnetic, cytocompatible, produced by an eco-friendly, cost effective, and scalable method [5,6], the novelty of BMNPs compared to traditional MNPs lies on changes on the size (which translates into magnetic properties), and surface properties of the former. In fact, the two more important advantages over synthetic MNPs are (1) BMNPs are larger than most MNPs, which maximizes their magnetic moment per particle, thus improving magnetic guidance [7] and, (2) MamC adsorbs (or even incorporates) into the outer layers of BMNPs, providing novel functional groups at the BMNP surface, which increases the versatility and efficiency of functionalization without the need of a post-production covering (otherwise required for MNPs) [5]. García Rubia at el. [5] demonstrated that BMNPs contain up to 4.5 wt% of MamC that gives them novel surface properties and provides functional groups that allow functionalization. In particular, the change in the isoelectric point (iep) of these BMNPs (pH 4.4) is noticeable compared to that of the inorganic magnetic nanoparticles (pH 7.0), which allows the electrostatic bonding between the BMNPs and the relevant molecule at physiological pH values, and the spontaneous release of such a molecule at acidic pH values [5]. It has been demonstrated that BMNPs are able to carry antitumor and antibacterial molecules such as DOXO, choline kinase inhibitors, oxaliplatin, AS-48, and signaling molecules as antibodies [5,6,8,9,10,11,12,13]. In fact, recent studies from our group demonstrate that BMNPs allow the combination of chemotherapy and magnetic hyperthermia in vitro [3] and in vivo in mice models, being that it is the most effective treatment against tumors compared to free drug [2]. BMNPs have been tested in vivo against localized breast cancer in mice models [8], and have been tested in vitro to mediate a directed chemotherapy (sometimes combined with hyperthermia) against other types of tumors [11,14], and even to treat bacterial infections [12]. However, a crucial factor, not explored until now, is the potential immune response that these nanoparticles may trigger following application. In fact, it is well known that the administration of some nanoparticles to the patients causes adverse effects such as inflammation, as a consequence of the release of pro-inflammatory mediators and ROS by immune cells [15,16,17,18,19,20,21,22]. Therefore, it is essential to check the effect of BMNPs on immune cell activity to understand whether they can be safely injected into the patients for therapeutic purposes.

IL-6 and TNF-α mediators secreted by monocytes and other immune cells are considered good indicators of the activation of an immune response [23,24,25]. Migrating monocytes at the sites of inflammation release these, and other, pro-inflammatory mediators to cooperate with the local innate immune response [26]. IL-6 and TNF-α activate the immune cells and play an essential role in the triggering of the systemic acute phase reaction characterized by fever, headache, changes in the sleep–wake cycle, anorexia, nausea, and emesis [23,24].

Moreover, the reactive oxygen species (ROS) that extravasated monocytes produce during inflammation [27] should also be taken into account. Whether or not the injection of BMNPs triggers ROS production needs to be investigated, as ROS are deeply involved in defenses against infections [28,29,30], but are also responsible for cellular oxidative damage, which could cause several diseases, such as cancer, hypertension, and neurological disorders [31].

In addition, the monocytes recruited into the tissues can differentiate into monocyte-derived dendritic cells (DCs) [32,33]. Immature (naïve) DCs (iDCs) sample and ingest foreign materials present in the extracellular environment and, subsequently, undergo a process of maturation (mDCs) which enables them to present the antigens to lymphocytes, thus activating the adaptive immune response [34,35,36]. In fact, nanoparticles have been previously reported to induce DC maturation [37,38,39,40], and, therefore the injection of these nanoparticles could lead to adverse consequences related to the stimulation of immune cells such as allergy or autoimmunity [41,42]. However, on the positive side, these nanoparticles could also be used as adjuvants to develop new vaccines [37,38,40]. Therefore, whether the injection of BMNPs may trigger the maturation of DC needs to be thoroughly evaluated to understand if and how BMNPs could be safely used for medical purposes.

Finally, a nanoparticle unable to activate immune cells and, therefore, generally considered biocompatible, could instead enhance the pro-inflammatory effect of some microorganisms or their derivatives [18,43,44,45] through a cooperative event named “cell priming” [46,47]. This implies that, if this apparently harmless nanoparticle is administered to the patient in the presence of other agents such as pathogenic fungi, bacteria, or viruses, it might cause unwanted dangerous effects as it can induce a hyper-responsiveness of leukocytes to the above-mentioned agents. Therefore, a possible cooperative effect between BMNPs and microbial molecules also needs to be investigated to assess the safety of the potential application of BMNPs as carriers for directed treatments against infections.

Based on these considerations, we investigated the effects of biomimetic magnetic nanoparticles (BMNPs) on the pro-inflammatory activity of human monocytes, including experiments on possible synergistic effects of these nanoparticles with molecules that mimic the interaction of cells with viruses, bacteria, or fungi. As a comparison, this effect was also studied on magnetic nanoparticles (MNPs) identically produced as BMNPs, but in the absence of MamC. Moreover, we analyzed whether MNPs and BMNPs were able to stimulate the maturation of monocyte-derived DCs or to interfere with the DC maturation process induced by cell stimulation with the bacterial lipopolysaccharide (LPS). We also evaluated the ability of monocytes and DCs to endocytose these two kinds of nanoparticles, to understand whether they could be useful tools to carry molecules such as drugs or antigens into the immune cells for therapeutic purposes.

## 2. Results

### 2.1. Characterization of MNPs and BMNPs

**Figure 1:**
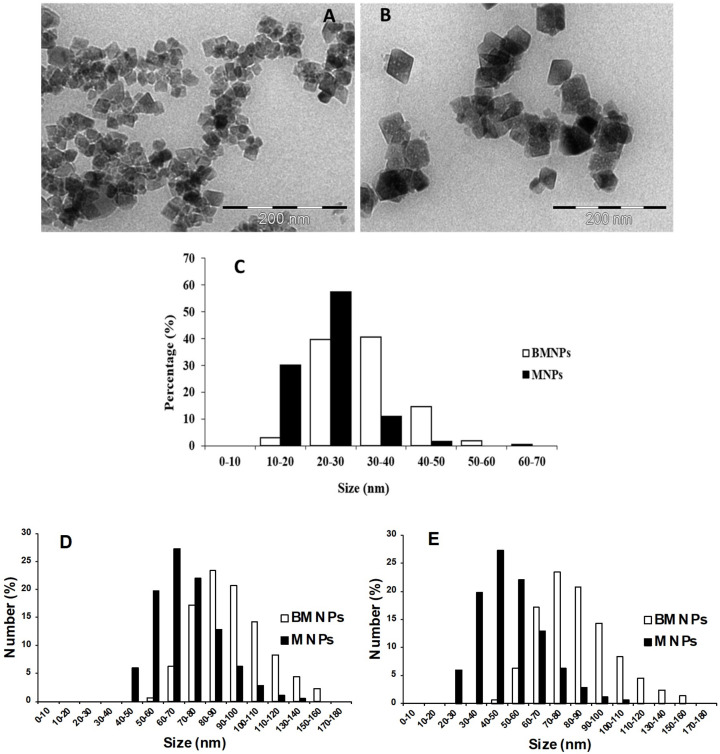
TEM images of MNPs (**A**) and BMNPs (**B**) (Scale bar = 200 nm.). (**C**) Size distribution of MNPs and BMNPs according to TEM analysis. Size distribution according to DLS data of BMNPs and MNPS resuspended in PBS pH 7.4 (**D**) and cell culture medium (**E**). All the DLS measurements were performed in triplicate.

Both MNPs and BMNPs were composed of >95% of magnetite, as shown by XRD analyses. Two-dimensional images of both MNPs and BMNPs show mainly rhombic shapes bounded by (111) crystal faces (Figure 1A). In BMNPs, rounded corners were very evident (Figure 1B), previously described as incipient (110) and (311) crystal faces [48]. A size histogram for MNPs and BMNPs (Figure 1C) shows that ~90% of MNPs displayed a size < 30 nm, while ~60% of BMNPs displayed a size >30 nm. The average size for MNPs was of 23 ± 6 nm, while that for BMNPs was of 32 ± 8 nm. The nanoparticle behavior in liquid suspension was also estimated by dynamic light scattering (DLS) measurements: MNPs show a size of 60 ± 28 nm with PDI 0.25 ± 0.15 once resuspended in PBS, and 43 ± 21 nm with PDI 0.44 ± 0.05 in the cell culture medium; BMNPs show a size of 88 ± 35 nm with PDI 0.34 ± 0.11 once resuspended in PBS, and 75 ± 30 nm with PDI 0.26 ± 0.12 in the cell culture medium (Table S1). The relatively high standard deviation of the size values is due to the polydispersity of the samples, as assessed by the PDI values. The size distribution of the nanoparticles follows a Gaussian curve (Figure 1D,E); the highest peak for MNPs in PBS shows that ~28% of the particles have a diameter between 60–70 nm; this peak is shifted to a range of 40–50 nm (~28% of the total population) when the particles are resuspended in the medium. Concerning BMNPs, the highest peak in PBS is between 80–90 nm with ~24 % of the particles in this range and also in this case, the peak moves to lower values (70–80 nm, ~24%) when the nanoparticles are in cell culture medium.

At pH 7.4 at which the in vitro cell experiments were performed, MNPs are neutral or slightly negatively charged (ζ-potential = −4 ± 1 mV), while BMNPs are negatively charged (ζ-potential = −32 ± 6 mV).

### 2.2. MNPs and BMNPs Derivatization by GFP Addition

In order to let the cellular uptake of the nanoparticles become evident by fluorescence microscopy, both MNPs and BMNPs were coupled to the green fluorescent protein (GFP) and the successful addition reaction was monitored by spectrofluorimetric analysis. Fluorescence emission spectra were recorded between 410 and 700 nm, exciting the samples obtained at 395 nm. Looking at the spectra for both MNPs and BMNPs coupled to GFP (Figure S1), the two characteristic peaks at approximately 450 nm and 510 nm of the fluorescent protein are evident [49]. Moreover, to verify if the fluorescence signal of GFP was attributable to the protein fraction bound to the nanoparticles, samples were visualized with a fluorescence microscope using a filter specific for GFP signal. From the images obtained from both MNPs and BMNPs coupled to GFP (Figure S2), the green fluorescence signal is coincident with the position of the nanoparticles with no residual fluorescence in the surrounding areas.

These data confirm the successful binding of GFP both on MNPs and BMNPs, maintaining the fluorescence property of the protein after the chemical reaction and making the magnetic nanoparticles become an efficient probe for fluorescence microscopy experiments aimed at verifying the nanoparticles cellular uptake.

### 2.3. Effect of MNPs and BMNPs on TNFα and IL-6 Release by Human Monocytes

**Figure 2:**
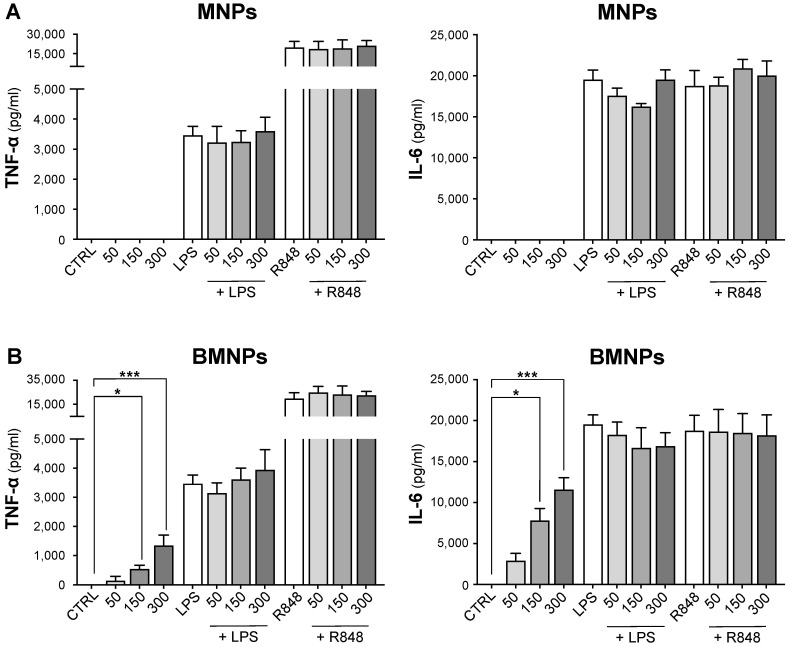
Effects of MNPs and BMNPs on TNF-α and IL-6 release by human monocytes. Monocytes were treated or not (CTRL) for 18 h with the indicated doses (μg/mL) of MNPs (**A**) or BMNPs (**B**) as well as with 100 ng/mL LPS or 5 μM R848 alone or in combination with MNPs or BMNPs. The release of TNF-α and IL-6 in culture supernatants was evaluated by ELISA assay. The results are expressed as the mean value + SEM of four independent experiments. * *p* < 0.05; *** *p* < 0.001 by Friedman or Kruskal–Wallis test followed by Dunn’s multiple comparison test.

MNPs did not trigger the release of TNF-α or IL-6 by monocytes (Figure 2A) whereas BMNPs induced the secretion of these chemical mediators in a dose-dependent manner, more evident at a dose of 300 μg/mL (Figure 2B). Nevertheless, this secretion was systematically much lower than that induced by exposure of monocytes to bacterial LPS, which binds TLR4, a pattern recognition receptors (PRR) activating the immune cells [50], or to R848, which binds TLR7 and TLR8, PRRs sensing viral RNA [51] (Figure 2B). Both LPS and R848 induced, as expected, TNF-α and IL-6 secretion by monocytes, independently of whether MNP or BMNPs were present or absent (Figure 2A,B). These results demonstrate that MNPs interact with human monocytes without activating the release of pro-inflammatory mediators (Figure 2A), whereas BMNPs significantly trigger the secretion of these mediators only at high doses, although this release appears lower than that induced by LPS or R848 (Figure 2B). Both MNPs and BMNPs are unable to cooperate with agonists of TLR4, TLR7, and TLR8 in the induction of TNF-α and IL-6 production indicating that they do not exert a “priming effect” on pro-inflammatory mediator secretion by monocytes (Figure 2A,B).

### 2.4. Effect of MNPs and BMNPs on ROS Production by Human Monocytes

**Figure 3:**
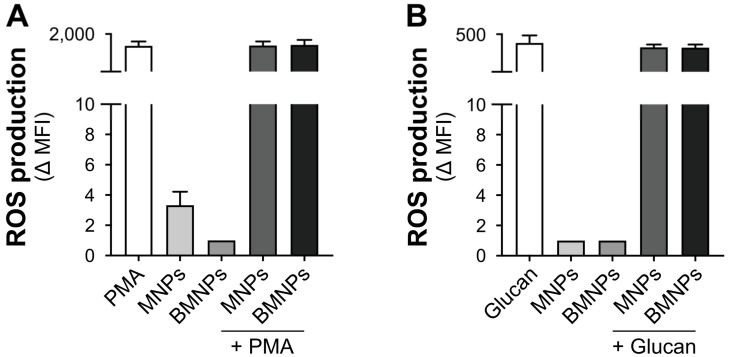
Effects of MNPs and BMNPs on ROS production by human monocytes. Monocytes were labeled with 5 µM ROS indicator probe CM-H2DCFDA and then treated for 30 min with 5 ng/mL PMA (**A**) or 10 μg/mL β-glucan (**B**) both in the absence or presence of 300 μg/mL MNPs or BMNPs, as well as with MNPs or BMNPs alone. ROS production was estimated by flow cytometry. Graphs show ROS production as Δ MFI values of CM-H2DCFDA probe. The values are expressed as the mean value + SEM of four independent experiments. Statistical analysis was performed by Friedman or Kruskal–Wallis test followed by Dunn’s multiple comparison test.

Results in Figure 3 show that, at a dose as high as 300 μg/mL, both MNPs and BMNPs were unable to activate ROS formation. Moreover, both MNPs and BMNPs were unable to enhance the PMA- or β-glucan-induced ROS production (Figure 3A,B).

### 2.5. BMNPs, but Not MNPs, Induced the Maturation of Monocyte-Derived DCs

**Figure 4:**
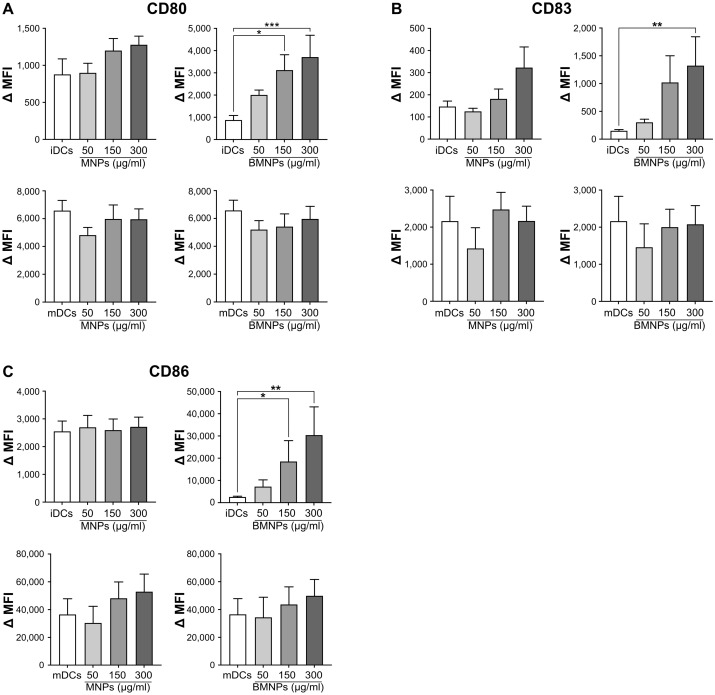
Effects of MNPs and BMNPs on monocyte-derived DC maturation. DCs cultured in the absence (iDCs) or presence (mDCs) of 100 ng/mL LPS were treated for 24 h with the indicated doses of MNPs or BMNPs. The expression of maturation markers CD80 (**A**), CD83 (**B**), and CD86 (**C**) was assessed by flow cytometry and shown as Δ MFI values. The results are expressed as the mean value + SEM of five independent experiments. * *p* < 0.05; ** *p* < 0.01; *** *p* < 0.001 by Friedman or Kruskal–Wallis test followed by Dunn’s multiple comparison test.

The monocytes have the ability to differentiate into DCs, which play a central role in the regulation of immune response [32,33]. An important feature of DCs is that following interaction with pathogens, they undergo a process of maturation which enables them to present the antigens to lymphocytes, thus activating the adaptive immune reaction [34,35,36,52]. To evaluate whether MNPs or BMNPs affect this important process, the monocytes were cultured with 50 ng/mL GM-CSF and 20 ng/mL IL-4 to induce their differentiation into iDCs [53]. Following this treatment, the cells were characterized by flow cytometry as CD1a^high^, CD80^−^, CD83^−^, and CD86^low^ indicating that differentiation to iDCs had occurred [54,55]. Subsequently the iDCs were cultured in the presence or absence of LPS, a powerful bacterial inducer of DC maturation [56] and treated with 50, 150, or 300 μg/mL MNPs or BMNPs: the expression of maturation markers CD80, CD83, and CD86 [57,58] was analyzed by flow cytometry. Interestingly, BMNPs, but not MNPs, significantly increased the expression of CD80, CD83, and CD86 in iDCs (Figure 4A–C). As expected, all these markers were up-regulated in LPS-treated mDCs (Figure 4A–C). However, both MNPs and BMNPs were unable to affect the LPS-induced CD80, CD83, and CD86 expression (Figure 4A–C). These findings indicate that BMNPs, but not MNPs, are able to induce DC maturation and therefore they could enhance the capacity of monocyte-derived DCs to present antigens. Furthermore, neither MNPs nor BMNPs should interfere with the DC maturation process induced by other agents, such as microbial molecules.

### 2.6. MNPs and BMNPs Uptake by Human Monocytes and DCs

**Figure 5:**
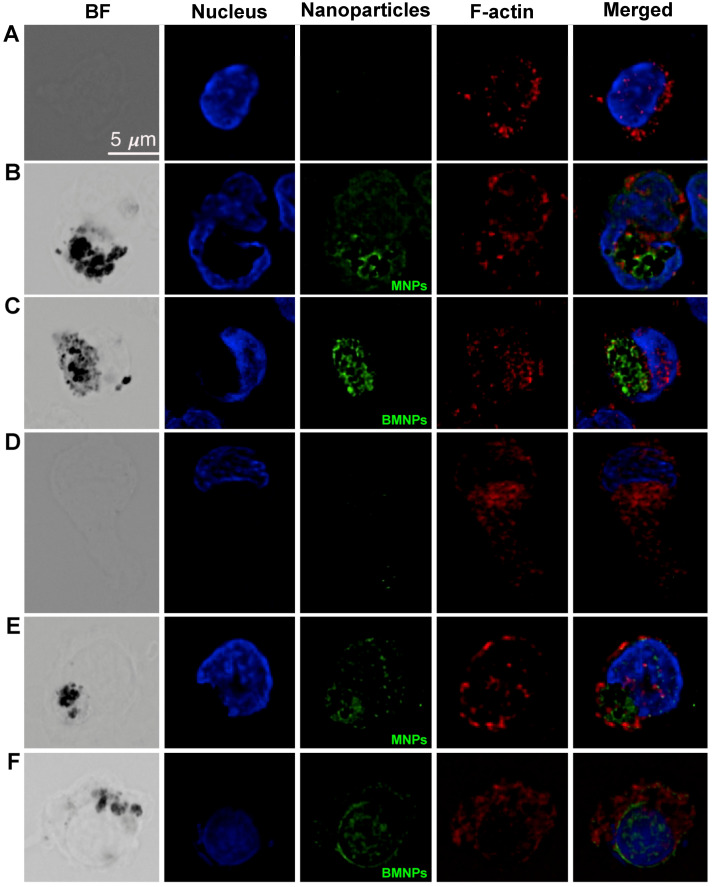
Internalization of MNPs and BMNPs by human monocytes and iDCs. Monocytes (panels (**A**–**C**)) and iDCs (panels (**D**–**F**)) were treated with 50 μg/mL GFP-conjugated MNPs (panels (**B**,**E**)) or BMNPs (panels (**C**,**F**)) for 2 h and the particle cellular uptake was visualized by wide-field deconvolution fluorescence microscopy. Untreated cells are illustrated in panels (**A**) and (**D**). Shown are six representative fields (top to bottom rows). From left to right, bright field (BF), DAPI (Nucleus), GFP (Nanoparticles), and SiR-actin (F-actin) individual channels are shown. On the far-right, merged channels are shown. Scale bar is 5 μm.

Figure 5 shows that both MNPs and BMNPs were efficiently internalized by human monocytes and iDCs after 2 h incubation. Three-dimensional scanning reconstruction and ortho-projection analysis showed that the particles were localized inside the cells (not shown). Of note, cells incubated in absence of nanoparticles (panel A and D) showed no green fluorescence, indicating that the detected fluorescence signal in the green channel (GFP) was specifically due to GFP-conjugated intracellular nanoparticles.

### 2.7. Effect of MNPs and BMNPs on the Viability of Human Monocytes and DCs

**Figure 6:**
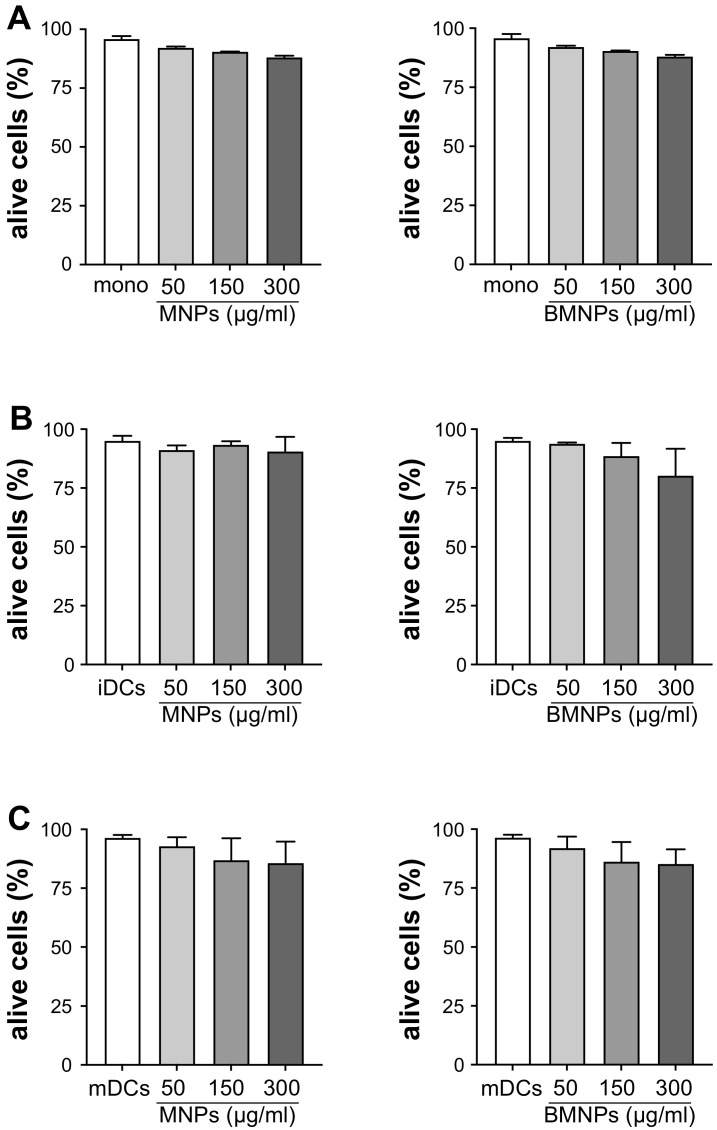
Effects of MNPs and BMNPs on the viability of human monocytes and DCs. Monocytes (mono) (**A**), immature DCs (iDCs) (**B**), and mature DCs (mDCs) (**C**) were challenged with 50, 150 or 300 μg/mL MNPs or BMNPs for 24 h and cell viability has been assessed by VYBRANT/SYTOX staining by flow cytometry. The results are expressed as the mean value of alive cells frequency + SEM of four independent experiments.

Figure 6A shows that neither MNPs nor BMNPs significantly affected monocytes’ viability. Identically, iDCs (Figure 6B) and mDCs (Figure 6C) viability was not altered by MNPs or BMNPs.

## 3. Discussion

Here, we report that BMNPs, but not MNPs, trigger the secretion of pro-inflammatory mediators by monocytes. According to our results, MNPs are 100% magnetite (Fe_3_O_4_), and, neither the mineral nor the ions that they are composed of induce an immune response. This result is in agreement with the conclusions of other authors, which demonstrate the biocompatibility of magnetite magnetic nanoparticles [59]. On the other side, BMNPs are also composed of magnetite as the only mineral phase, according to XRD analyses, but these nanoparticles do trigger pro-inflammatory activities at the higher doses. This effect could be due to the fact that, while MNPs were synthesized chemically in the absence of any bacterial derivatives, BMNPs were synthesized in the presence of MamC from *Magnetococcus marinus*, and it has been shown that this protein is attached to (or even incorporates in) BMNPs [4]. These authors concluded that MamC is attached to BMNPs thanks to their results of thermogravimetric analysis of the nanoparticles and ζ-potential. Therefore Garcia-Rubia et al. [4] concluded that MamC was strongly attached (if not incorporated) to the surface of the magnetite core. Results in the present paper agree with this conclusion, as, except for MNPs, BMNPs trigger the immune response at high doses following their injection.

However, the immune response could also be triggered by bacterial derivatives attached to MamC. This protein was expressed as recombinant protein in *Escherichia coli* and purified by FPLC. However, and although gel electrophoresis of the purified MamC shows a size of 17.4 KDa, corresponding to the expected size of the protein (17,46 KDa, *ExPAsy Server*; Figure S3), and although BMNPs were sterilized, the presence of trace bacterial derivatives able to stimulate immune cells cannot be ruled out.

Other parameters related to cell uptake can also influence immune response. In vitro studies present in the literature report nanoparticles with a size range of 10–60 nm as the best endocytosed by cells [60]. Once our nanoparticles are resuspended in the cell growth medium, most of them form clusters of 2–3 units, as it is evident from the DLS data reported in Table S1. Analyzing the size distribution data reported in Figure 1D,E, we can infer that the percentage of nanoparticles with a size ≤ 60 nm is increased for MNPs from ~54.8% when in PBS to ~89.7% when they are in the cell culture media. The same tendency is seen also for BMNPs, where the percentage of nanoparticles with a size ≤ 60 nm changes from ~7.4% when in PBS to ~25.3% when in the cell culture medium used for in vitro experiments. We can assume that also the different level of aggregation between MNPs and BMNPs could affect the interaction with cells and differentially activate their response [61]. In addition, surface charge and hydrophobic interactions are important to determine the ability of the nanoparticles to interact with cells. As BMNPs are charged and expose the charged (and hydrophobic) MamC domains, thanks to the presence of MamC at their surface [5], their interaction with the cells might be favored compared to that of MNPs, which present an almost zero charge and do not have any hydrophobic groups at their surface.

The activation of the immune response by BMNPs was not entirely unexpected, because it is well known that immune cells undergo activation upon interaction with several nanoparticles [15,16,17,19,20,21], including some which are generally considered safe and biocompatible such as PLGA nanoparticles [18,22]. In particular, some magnetic iron oxide nanoparticles have been shown to increase ROS and pro-inflammatory cytokine release in THP 1 macrophages [62] and to enhance the levels of IL-2, interferon-γ and IL-10 in mice peripheral blood [63].

Moreover it should be emphasized that (1) BMNPs only significantly activated the secretion of pro-inflammatory mediators by monocytes at very high doses, especially >300 μg/mL, so this study poses the highest dose for BMNPs to be injected to avoid immune cell stimulation, and (2) the secretion of TNFα and IL-6 induced by BMNPs was much lower than that elicited by the immune cell activators R848 and LPS, suggesting that, even at these high concentrations (>300 μg/mL), BMNPs would cause only a mild inflammatory effect. Our results are in agreement with studies of the effects of other iron oxide nanoparticles on immune cells, for instance, dextran-coated iron oxide nanoparticles did not activate pro-inflammatory cytokine production or superoxide anions in human monocyte–macrophages [64], and these particles induced only a slight IL-1 production in mouse peritoneal macrophages [65].

Furthermore, neither MNPs or BMNPs primed pro-inflammatory mediator release by monocytes upon R848- and LPS-stimulation and neither of them induced ROS production nor did they affect that triggered by β-glucan or PMA. Therefore, both MNPs and BMNPs are unable to activate pathways that cooperate in (1) the induction of TNFα and IL-6 release with signals originated from TLR7, TLR8, and TLR4, and (2) activation of ROS production with β-glucan- or PMA- triggered events. This is important because a synergy between nanoparticles and microbial molecules is not an uncommon event, although it is usually scantly taken into consideration. In particular it has been reported that the microbial chemoattractant N-formyl-methionyl-leucyl-phenylalanine (fMLP) synergized with ORMOSIL nanoparticles to trigger the release of cytokines by human leukocytes [43]. Additionally, porous silicon-TiO_2_ microparticles increased the LPS-induced IL-12 and TNF-α secretion by human DCs [44]. Moreover, PLGA nanoparticles increased IL-12, TNFα, and IL-6 secretion by R848-stimulated DCs [45] and enhanced β-glucan-induced O_2_^−^ production in human monocytes [18]. Therefore our finding that both MNPs and BMNPs did not cooperate with three stimuli such as β-glucan [66,67], R848 [51], and LPS [50,56], that mimic the natural interaction between immune cells and pathogenic fungi, viruses, and bacteria, as well as with PMA, a molecule structurally related to the natural NOX2 NADPH oxidase activator diacylglycerol [68,69] suggests that both magnetic nanoparticles could be good candidates for medical applications.

Nevertheless, the finding that incubation of monocyte-derived DCs with BMNPs led to up-regulation of molecules expressed during DC maturation (Figure 4) suggests that BMNPs could be exploited as adjuvants in the formulation of new vaccines. The induction of DC maturation is, in fact, of great importance in developing new vaccination strategies [37,38,39,40,70,71]. However, it must be considered that an activation of DCs could also promote pathological events such as hypersensitivity and autoimmunity [41,42], so further investigations are required to assess whether BMNPs can be safely used as adjuvants. The result that both MNPs and BMNPs did not affect the LPS-induced expression of CD80, CD83, and CD86 indicates that these nanoparticles might not hinder or increase the maturation of DCs induced by other stimulatory molecules: this result further underlines the biocompatibility of those nanoparticles.

High doses of MNPs and BMNPs did not significantly damage the monocytes and both iDCs and mDCs, as demonstrated by the experiments assessing cell viability reported in this paper (Figure 6). These results combined with the findings that both MNPs and BMNPs were very efficiently internalized by human monocytes and DCs, indicate that these nanoparticles can be considered as useful vehicles to carry molecules such as drugs or antigens inside these cells to regulate their activity and consequently, the immune response against tumors or infectious diseases.

It is worth pointing out that monocytes are part of the so-called professional phagocytes accomplishing phagocytosis with high efficiency, and the engulfment of foreign materials usually results in their activation [72]: our findings that MNPs and BMNPs were greedily endocytosed by monocytes without a substantial production of cytokines and ROS further indicate that these particles did not importantly disturb the functionality of these cells and therefore they should not activate important unwanted inflammatory side-effects when used in therapy.

## 4. Materials and Methods

### 4.1. Synthesis and Characterization of MNPs and BMNPs

Both MNPs and BMNPs were synthesized as previously described by Jabalera et al. (2019) [73]. Briefly, MamC was cloned, expressed, and purified under denaturing conditions following the protocol of Valverde-Tercedor et al. (2015) [7]. Purification was carried out using a HiTrap chelating HP column (GE Healthcare, Chicago, IL, USA) in an AKTA Prime Plus FPLC system (GE Healthcare). Both types of nanoparticles, MNPs and BMNPs, were synthetized inside an anaerobic COY chamber from the following master solution: Fe(ClO_4_)_2_ (2.78 mM), NaHCO_3_ (3.5 mM), Na_2_CO_3_ (3.5 mM), and FeCl_3_ (5.56 mM), at pH 9, to which 10 μg/mL of MamC were added in the case of BMNPs. The formation of the nanoparticles occurred in free-drift experiments for 30 days at 25 °C and 1 atm total pressure. The precipitates were magnetically concentrated and rinsed with oxygen-free MilliQ water four times.

The morphology and size of the precipitates were studied by transmission electron microscopy (TEM, LIBRA 120 PLUS, Carl Zeiss SMT, Oberkochen, Germany) on ultrathin sections. Magnetic nanoparticles were resuspended in ethanol and embedded in Embed 812 resin. Ultrathin sections (50−70 nm) were prepared using a Reichert Ultracut S microtome (Leica Microsystems GmbH, Wetzlar, Germany), and deposited onto copper grids. The crystal size was measured on 1000 nanoparticles per experiment using ImageJ 1.47. Powder X-ray diffraction (XRD) analysis was carried out on lyophilized samples with an Xpert Pro X-ray diffractometer (Malvern Panalytical, Malvern, UK). ζ-potential of both MNPs and BMNPs at the pH at which the in vitro cell experiments were performed (pH = 7.4) was calculated from electrophoretic mobility measurements carried out in a Zetasizer Nano-ZS (Malvern Instruments, Malvern, UK) at 25 °C as detailed by García Rubia et al. (2018). The size of both MNPs and BMNPs was estimated also by dynamic light scattering using the same apparatus. Each sample was re-suspended in PBS or in cell culture medium to a final concentration of 0.3 mg/mL and measured in triplicate with the sample cell temperature fixed at 25 °C. Data were collected and analyzed by the ZetaSizer software Version 7.12.

### 4.2. Preparation of GFP-Conjugated MNPs and BMNPs

The green fluorescent protein used in the nanoparticle derivatization process was obtained as a subproduct from the purification of the recombinant Boletus edulis lectin (BEL) β-trefoil used in other experiments [74]. *E. coli* BL21 (DE3) strain was transformed with the plasmid pWaldo-GFP [75] used as a vector of the BEL β-trefoil coding sequence [76], providing a C-terminal GFP and an hexahistidine tag (His-tag) useful for the subsequent purification steps, separated from the gene of interest by a *Tobacco etch virus* (TEV) protease cleavage site. Cells were grown at 37 °C until an OD_600_ of 0.8 in LB media and the protein expression was induced adding 0.25 mM IPTG to the medium and shacking overnight at 20 °C. Harvested cells were lysed by sonication, collected by centrifugation and the soluble fraction was loaded onto a Nickel–Sepharose column; the purification of the expressed protein was carried out applying a linear gradient of imidazole. GFP was obtained adding TEV protease to the protein solution, previously dialyzed against 20 mM Tris-HCl pH 7.5, 500 mM NaCl, and flowing the obtained protein mixture again through a Nickel–Sepharose column: the histidine-tag-free BEL β-trefoil does not interact with the metal in the column where instead GFP provided by the His-tag does. Pure GFP was eluted adding a concentration of 350 mM imidazole to the previous buffer, dialyzing the eluate against PBS (0.137 M NaCl, 2.7 mM KCl, 10 mM Na_2_HPO_4_, 1.8 mM KH_2_PO_4_, pH 7.4) after concentration and the protein solution was then sterilized on a 0.2 µm syringe filter.

The immobilization of GFP on both MNPs and BMNPs was obtained via 1-ethyl-3-(3- dimethylaminopropyl) carbodiimide (EDC)/N-hydroxysuccinimide (NHS) reaction: 5 mg of either MNPs or BMNPs were resuspended in 1 mL of 50 mM MES free oxygen buffer (pH 5) and activated with EDC (0.1 M) and NHS (0.7 M); the reaction solution was stirred for 1 h at 20 °C; GFP was added to the suspension at a final concentration of 30 µM and incubated for 24 h at room temperature. The coupling reaction was stopped by the addition of Tris 0.1 M, and the GFP-derived nanoparticles were magnetically recovered from the reaction mixture using a permanent magnet. After three wash steps with PBS buffer, the successful immobilization was detected acquiring fluorescence spectra (FP-8200, Jasco, Easton, MD, USA), exciting the samples at 395 nm (excitation bandwidth 5 nm, emission bandwidth 5 nm, response 1 sec, and sensitivity high), and confirming these data by imaging using an EVOS FLoid imaging system (Thermo Fisher Scientific, Winsford, UK); samples were diluted 12 times in PBS before the analysis.

### 4.3. Monocytes and DCs Preparation and Culture

After written informed consent and upon approval of the ethical committee (Prot. N. 5626, 2 February 2012; Prot. n. 57182, 16 October 2019), buffy coats from the venous blood of normal healthy volunteers were obtained from the Blood Transfusion Centre of the University of Verona. Peripheral blood mononuclear cells were isolated by Ficoll-Paque^TM^ PLUS (Cytiva, Uppsala, Sweden) density gradient and human CD14^+^ monocytes were isolated from PBMCs by anti-CD14 microbeads kit (130–050-201 Miltenyi Biotec, Bergisch Gladbach, Germany). The purity of CD14^+^ cells was always greater than 98%, as determined by flow cytometry. To generate immature DCs, monocytes were cultured at 37 °C, 5% CO_2_ at 1 × 10^6^/^mL^ in 6-well tissue culture plates (Corning Incorporated, NY, USA) in RPMI-1640 (Corning) supplemented with 10% FBS (<0.5 EU/mL; Sigma-Aldrich (St. Louis, MO, USA) and stimulated with 50 ng/mL GM-CSF and 20 ng/mL IL-4 (Miltenyi Biotec). After 5 days, non-adherent iDCs were harvested and characterized by flow cytometry as CD1a^high^, CD80^−^, CD83^−^, and CD86^low^. To induce cell maturation, DCs were treated with 100 ng/mL LPS ultrapure, *E. coli* 0111: B4 strain, (InvivoGen, San Diego, CA, USA) for 24 h, then harvested and characterized by flow cytometry as CD80^+^, CD83^+^, and CD86^high^.

### 4.4. Quantification of Cytokine Production

ELISA development kits purchased from Mabtech (Nacka Strand, Sweden) were used to assay the protein levels of TNF-α (range 4–400 pg/mL) and IL-6 (range 10–1000 pg/mL) in the cell culture supernatant, according to the manufacturer’s instructions. Briefly, monocytes were treated with 50, 150, or 300 μg/mL MNPs or BMNPs as well as with 100 ng/mL LPS or 5 μM R848 (InvivoGen, San Diego, CA, USA) alone or in combination with MNPs or BMNPs. After 18 h, the supernatants were collected and processed as previously described [45].

### 4.5. Quantification of ROS Production

Monocytes were labeled with 5 µM ROS indicator 5-(and-6)-chloromethyl-2′,7′-dichlorodihydrofluorescein diacetate, acetyl ester (CM-H2DCFDA) (C6827 Molecular Probes™, Thermo Fisher Scientific, Waltham, MA, USA) for 15 min at 37 °C. Subsequently, monocytes were treated for 30 min with 5 ng/mL PMA or 10 μg/mL β-glucan from baker’s yeast (Sigma-Aldrich, St. Louis, MO, USA) both in the absence or presence of 300 μg/mL MNPs or BMNPs, as well as with MNPs or BMNPs alone, and then ROS production was estimated by flow cytometry. The delta median fluorescence intensity (Δ MFI), relative to ROS production, was obtained by subtracting the MFI of CM-H2DCFDA-labeled cells (control) from the MFI of stimulated CM-H2DCFDA-labeled cells.

### 4.6. Evaluation of DC Maturation Markers Expression

DCs were cultured in the absence or presence of 100 ng/mL LPS and treated for 24 h with 50, 150 or 300 μg/mL MNPs or BMNPs. The expression of maturation markers CD80, CD83, and CD86 was assessed by flow cytometry. Typically, 2 × 10^5^ cells were resuspended in 50 μL of PBS (Corning) containing 2% FBS (Sigma-Aldrich) and 2 mM EDTA (Sigma-Aldrich) (staining buffer), and subsequently incubated for 30 min at 4 °C with 5% human serum (Sigma-Aldrich). Cells were then stained for 30 min on ice using the following mouse anti-human antibodies: CD1a-FITC (HI149), CD80-PE (2D10), CD83-PE (HB15), and CD86-PE (IT2.2) (Biolegend, San Diego, CA, USA), and CD14-VioBlue (TÜK4) (Miltenyi Biotec).

For immunophenotypic characterization, data from 150,000 events were acquired by MACSQuant 16 Analyzer (Miltenyi Biotec), while data analysis was performed using FlowJo software version 10.7.1 (BD Biosciences, Heidelberg, Germany). Phenotypic analysis was performed on alive cells identified as Sytox^TM^ Advanced Dead Cell Stain Kit (Thermo Fisher Scientific) negative cells. The delta median fluorescence intensity (Δ MFI) relative to each marker was obtained by subtracting either the MFI of the correspondent isotype control or the cell autofluorescence (revealed as Fluorescence Minus One, FMO, control) from the MFI of the specific antibody.

### 4.7. Immunofluorescence and Microscopy Analysis

Monocytes and DCs were treated with 50 μg/mL of GFP-conjugated MNPs or BMNPs for 2 h. After washing with PBS, cells were directly stained with 50 nM SiR-actin (SC001, Spirochrome AG/Tebu-Bio srl, Milano, Italy), a fluorogenic cell that is a permeable and highly specific probe for F-actin, for 2 h. Cells were then fixed with 0,4% paraformaldehyde (Sigma-Aldrich) for 30 min at room temperature and incubated for 10 min with DAPI (Sigma-Aldrich) to stain nuclei. Coverslips were mounted with Fluoro Gel with DABCO^TM^ (Electron Microscopy Sciences, Hatfield, PA, USA) on microscopy slide. After complete drying, images were acquired with a wide field Zeiss AxioOberver 7 deconvolution microscopy setting (Carl Zeiss, Oberkochen, Germany), equipped with Colibri 7 fluorescent LED illumination, motorized 3D scanning stage and ORCA-Flash4.0 V3 Digital CMOS camera (Hamamatsu Inc., Hamamatsu, Japan), set at an 8 output bit depth. Pixels of 1024 × 1024 ROIs were acquired with a 40× Plan Apochromatic objective (AN 0.96). Each field was acquired with triple fluorescent light illumination (385/30 nm ex. for DAPI, 646/30 nm ex. for Cy5-SiR-actin and 488-30 for GFP. Automatic 3D image scanning was according to the Nyquist-Shannon sampling theorem, by using the inline ZEN 2.6 Nyquist Calculator. 3D scans were, then, processed with Zeiss ZEN 3.5 by applying the constrained iterative deconvolution algorithm with the Zeiss Deconvolution (DCV) advanced module (Carl Zeiss).Image deconvolution was achieved by applying the Constrain Iterative algorithm. Spectral linear unmixing was, finally, applied to remove overlapped spectral components and background noise. Deconvolved and unmixed 3D stacks were rendered and analyzed with the ZEN 3.5 Arivis 3D module (Carl Zeiss).

### 4.8. Cell Viability Evaluation

Monocytes, iDCs, and mDCs, were treated with 50, 150, or 300 μg/mL MNPs or BMNPs for 24 h and cell viability was assessed by VYBRANT/SYTOX (Thermo Fisher Scientific) staining. Cells (2 × 10^5^) were centrifuged at 400× *g* for 5 min to remove the medium, and then suspended in 100 μL Hank’s balanced salt solution (HBSS) buffer containing 5 µM Vybrant™ DyeCycle™ Violet stain and 5 μM SYTOX™AADvanced™ Dead cell stain kit (Thermo Fisher Scientific). Cells were then put on ice for 30 min, protected from light. Then cells were washed and suspended in 100 μL HBSS buffer and sample fluorescence was measured by MACSQuant16 Analyzer (Miltenyi Biotec). Cell viability was defined as the percentage of cells that were double negative for both stains (Vybrant™/Sytox™, respectively).

### 4.9. Statistical Analysis

The comparison of variables was performed using an unpaired two-tailed Mann–Whitney test (for comparison between two groups) and either the Friedman or the Kruskal–Wallis test followed by Dunn’s multiple comparison test (for comparison between three or more groups).

*p*-values of less than 0.05 were considered significant and asterisks indicate significant increases: * = *p*-value < 0.05; ** = *p*-value < 0.01; *** = *p*-value < 0.001.

Graphs were elaborated using GraphPad Prism Version 9 software (GraphPad Software Inc., San Diego, CA, USA).

## 5. Conclusions

The assessment of the biocompatibility of nanoparticles that could be used for medical purposes requires investigations on the consequences of their interaction with the cells of the organism, and in particular, with immune cells. Owing to the essential role of monocytes and DCs in the regulation of the inflammation and immune reaction, investigations on the effect of nanoparticles on these cells could be useful to determine the safety of their use in clinical practice. The relevance of the results reported here consists of highlighting that BMNPs, at doses < 150 μg/mL, did not substantially perturb the functions of monocytes. Only at a high dose of ≥300 μg/mL, BMNPs produced an important activation of the immune response, which, in any case, was lower than that triggered by stimuli such as β-glucan, R848, and LPS that mimic the natural interaction between immune cells and pathogenic fungi, viruses, and bacteria. Importantly, BMNPs were rapidly endocytosed by both monocytes and DCs without damaging these cells, indicating that these nanoparticles could be used as vehicles to carry therapeutic molecules inside the immune cells. Moreover, BMNPs were able to induce monocyte-derived DC maturation. Although in vivo studies would be necessary to ensure that the evaluated nanomaterials are biocompatible, our data provide a preliminary demonstration that BMNPs are nanostructures that can be considered as good candidates for use in medicine.

## Data Availability

No new data were created or analyzed in this study. Data sharing is not applicable to this article.

## Supplementary Materials

The following supporting information can be downloaded at: https://www.mdpi.com/article/10.3390/ijms24021358/s1.
