# Supplementary material for: Effects of Magnetic Nanoparticles on the Functional Activity of Human Monocytes and Dendritic Cells
Source: Int J Mol Sci. 2023 Jan 10;24(2):1358. doi: 10.3390/ijms24021358 (PMC9864373; doi:10.3390/ijms24021358)
Supplement: Supplementary file 1 [file ijms-24-01358-s001.zip › ijms-2093882-supplementary.pdf]

# Effects of Magnetic Nanoparticles on the Functional Activity of Human Monocytes and Dendritic Cells

**Marta Donini [1,†], Francesca Pettinella [1,†], Giorgia Zanella [2], Salvatore Calogero Gaglio [2], Carlo Laudanna [1], Monica Jimenez-Carretero [3], Concepcion Jimenez-Lopez [3,*], Massimiliano Perduca [2,*,‡] and Stefano Dusi [1,‡]**

1 Section of General Pathology, Department of Medicine, University of Verona, Strada Le Grazie 8, 37134 Verona, Italy
2 Department of Biotechnology, University of Verona, Strada Le Grazie 15, 37134 Verona, Italy
3 Department of Microbiology, Faculty of Sciences, University of Granada, 18071 Granada, Spain
* Correspondence: cjl@ugr.es (C.J.-L.); massimiliano.perduca@univr.it (M.P.)
† These authors contributed equally to this work.
‡ These authors equally contributed to conceptualization and supervision.

**Table S1.** Dynamic Light Scattering parameters for MNPs and BMNPs resuspended in PBS or cell culture medium.

| **PBS** | **Size (nm)** | **PDI** |
|---|---|---|
| BMNPS | 88 ± 35 | 0.34 ± 0.11 |
| MNPS | 60 ± 27 | 0.25 ± 0.15 |
| | | |
| **Medium** | **Size (nm)** | **PDI** |
| BMNPS | 75 ± 30 | 0.26 ± 0.12 |
| MNPS | 43 ± 21 | 0.44 ± 0.05 |

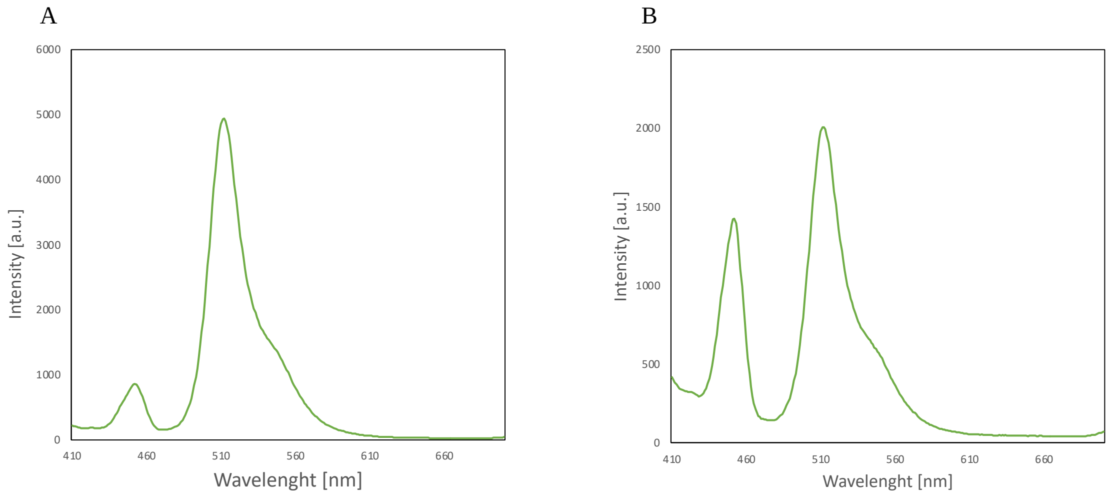


**Figure S1.** Fluorescence spectra for MNPs (A) and BMNPs (B) coupled to GFP. Data were acquired using a Jasco FP-8200 fluorescence spectrometer, exciting the samples at 395nm (excitation bandwidth 5 nm, emission bandwidth 5 nm, response 1 sec, and sensitivity high). The double peak present in both analyzed nanoparticles denotes GFP.

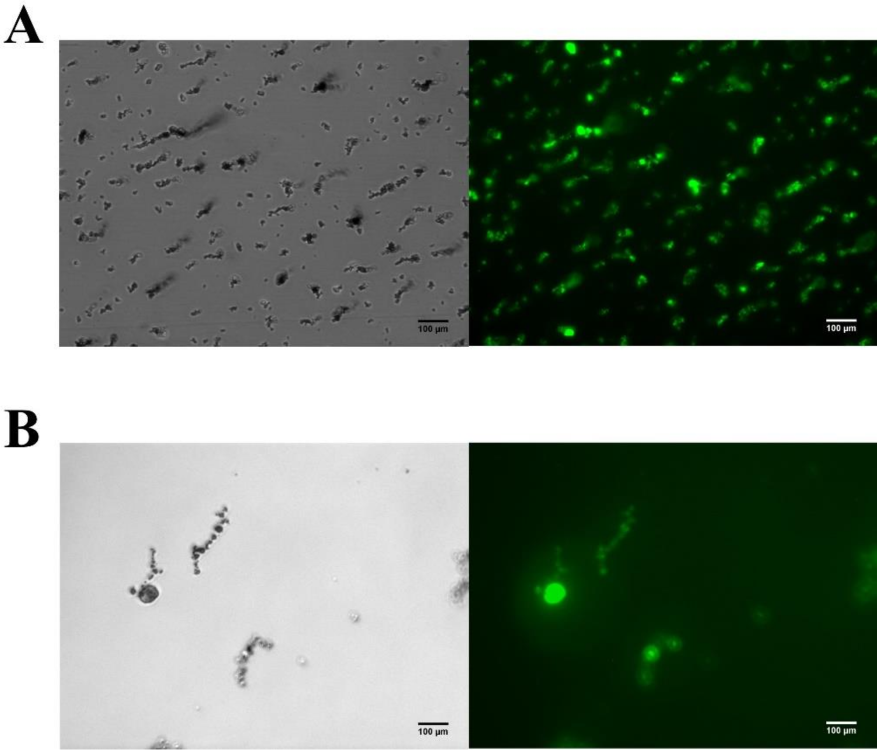


**Figure S2.** Fluorescence microscopy representative images for MNPs (A) and BMNPs (B) coupled to GFP. Images were acquired using an EVOS FLoid imaging System with a 40X magnification and diluting 12 times with PBS the stock samples.

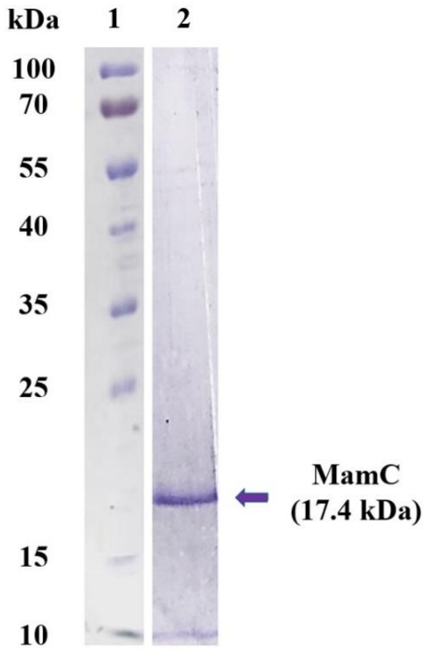


**Figure S3.** Gel electrophoresis of the purified MamC.
